# Histone deacetylase inhibitors modulate metalloproteinase gene expression in chondrocytes and block cartilage resorption

**DOI:** 10.1186/ar1702

**Published:** 2005-02-22

**Authors:** David A Young, Rachel L Lakey, Caroline J Pennington, Debra Jones, Lara Kevorkian, Dylan R Edwards, Timothy E Cawston, Ian M Clark

**Affiliations:** 1 School of Biological Sciences, University of East Anglia, Norwich, UK; 2 Department of Rheumatology, University of Newcastle-upon-Tyne, Newcastle-upon-Tyne, UK; 3 Department of Rheumatology, University of Newcastle-upon-Tyne, Newcastle-upon-Tyne, UK

## Abstract

Cartilage destruction in the arthritides is thought to be mediated by two main enzyme families: the matrix metalloproteinases (MMPs) are responsible for cartilage collagen breakdown, and enzymes from the ADAMTS (a disintegrin and metalloproteinase domain with thrombospondin motifs) family mediate cartilage aggrecan loss. Many genes subject to transcriptional control are regulated, at least in part, by modifications to chromatin, including acetylation of histones. The aim of this study was to examine the impact of histone deacetylase (HDAC) inhibitors on the expression of metalloproteinase genes in chondrocytes and to explore the potential of these inhibitors as chondroprotective agents. The effects of HDAC inhibitors on cartilage degradation were assessed using a bovine nasal cartilage explant assay. The expression and activity of metalloproteinases was measured using real-time RT-PCR, western blot, gelatin zymography, and collagenase activity assays using both SW1353 chondrosarcoma cells and primary human chondrocytes. The HDAC inhibitors trichostatin A and sodium butyrate potently inhibit cartilage degradation in an explant assay. These compounds decrease the level of collagenolytic enzymes in explant-conditioned culture medium and also the activation of these enzymes. In cell culture, these effects are explained by the ability of HDAC inhibitors to block the induction of key MMPs (e.g. MMP-1 and MMP-13) by proinflammatory cytokines at both the mRNA and protein levels. The induction of aggrecan-degrading enzymes (e.g. *ADAMTS4*, *ADAMTS5*, and *ADAMTS9*) is also inhibited at the mRNA level. HDAC inhibitors may therefore be novel chondroprotective therapeutic agents in arthritis by virtue of their ability to inhibit the expression of destructive metalloproteinases by chondrocytes.

## Introduction

Articular cartilage is made up of two main extracellular-matrix (ECM) macromolecules, namely, type II collagen and aggrecan (a large, aggregating proteoglycan) [1,2]. The type II collagen scaffold endows the cartilage with its tensile strength, while the aggrecan, by virtue of its high negative charge, draws water into the tissue, swelling against the collagen network, and enabling the tissue to resist compression. Quantitatively more minor components (e.g. types IX, XI, and VI collagens; biglycan; decorin; cartilage oligomeric matrix protein; etc.) also have important roles in controlling matrix structure and organisation [2].

Normal cartilage ECM is in a state of dynamic equilibrium, with a balance between synthesis and degradation. For the degradative process, the major players are metalloproteinases that degrade the ECM, and their inhibitors. Pathological cartilage destruction can therefore be viewed as a disruption of this balance, favouring proteolysis.

The matrix metalloproteinases (MMPs) are a family of 23 enzymes in man that facilitate turnover and breakdown of the ECM in both physiology and pathology. The MMP family contains the only mammalian proteinases that can specifically degrade the collagen triple helix at neutral pH. These include the 'classical' collagenases – MMP-1, -8, and -13 – and also MMP-2 and MMP-14 (which cleave the triple helix with less catalytic efficiency). The enzyme(s) responsible for cartilage collagen cleavage in the arthritides remains open to debate [3].

A second group of metalloproteinases, the ADAMTS (a disintegrin and metalloproteinase domain with thrombospondin motifs) family, consists of 19 members, including the so-called 'aggrecanases', currently ADAMTS-1, -4, -5, -8, -9, and -15 [4-7]. Current data support the hypothesis that aggrecanases are active early in the disease process, with later increases in MMP activity (several MMPs can also degrade aggrecan), but the exact enzyme(s) responsible for cartilage aggrecan destruction at any stage in arthritis is unclear [3,8,9].

A family of four specific inhibitors, the tissue inhibitors of metalloproteinases (TIMPs), has been described. TIMPs are endogenous inhibitors of MMPs and potentially of ADAMTSs [10]. The ability of TIMP-1 to -4 to inhibit active MMPs is largely promiscuous, though a number of functional differences have been uncovered. TIMP-3 appears to be the most potent inhibitor of ADAMTSs, for example, with a subnanomolar *K*_i _against ADAMTS-4 [3].

Metalloproteinase activity is regulated at multiple levels, including gene transcription. However, the role of chromatin modification, and in particular acetylation, is little researched in the metalloproteinase arena. The packaging of eukaryotic DNA into chromatin plays an important role in regulating gene expression. The DNA is wound round a histone octamer consisting of two molecules each of histones H2A, H2B, H3, and H4, to form a nucleosome [11]. This unit is repeated at intervals of approximately 200 base pairs, with histone H1 associating with the intervening DNA. Nucleosomes are generally repressive to transcription, hindering access of the transcriptional apparatus [11]. However, two major mechanisms modulate chromatin structure to allow transcriptional activity: ATP-dependent nucleosome remodellers such as the Swi/Snf complex [12,13]; and the enzymatic modification of histones, via acetylation, methylation, and phosphorylation [14-16].

Acetylation by histone acetyltransferases occurs on specific lysine residues on the N-terminal tails of histones H3 and H4. This neutralisation of positive charge leads to a loosening of the histone:DNA structure, allowing access of the transcriptional machinery; furthermore, the acetyl groups may associate with and recruit factors containing bromodomains [11]. Many transcriptional activators or coactivators have (or recruit) histone acetyltransferase activity, giving a mechanism whereby acetylation can be targeted at specific gene promoters [15,16]. Conversely, histone deacetylases (HDACs) have also been characterised. Hypoacetylation of histones associates with transcriptional silence, and several transcriptional repressors and corepressors have been identified that have (or recruit) HDAC activity [17-19]. Nonhistone substrates of histone acetyltransferases have also been described, for example, p53, E2F, nuclear factor κB, Sp3, and c-Jun [20,21].

There are two families of HDACs, the NAD^+^-dependent, so-called SIR2 family (sometimes called class III HDACs), and the classical HDAC family. The classical HDACs can be grouped into three classes (I, II, and IV) based on phylogeny [22]. Class I HDACs (HDAC1, 2, 3, and 8) are related to yeast RPD3, and class II HDACs (HDAC4, 5, 6, 7, 9, and 10) are more closely related to yeast HDA1 [17]. HDAC11 alone represents class IV, and HDAC11-related proteins have been described in all eukaryotic organisms other than fungi [22]. Trichostatin A (TSA) and sodium butyrate (NaBy), are HDAC inhibitors [23,24] with a broad spectrum of activity against class I and II HDACs, but not the SIR2 family. Addition of these reagents to cells should therefore block histone deacetylation and result in increased acetylation of histones on susceptible genes. The prediction would be that this would lead to an increase in gene expression, and this is largely borne out experimentally. However, there are many instances of HDAC inhibitors acting as repressors of gene expression [25-29].

HDAC inhibitors have potent antiproliferative and pro-apoptotic activities in cancer cells and this has led to the development of specific inhibitors for cancer chemotherapy. Such compounds are currently in both preclinical development and clinical trials [30].

Two recent reports demonstrate that HDAC inhibitors modulate gene expression in synovial cells [31]. In an animal model of rheumatoid arthritis (adjuvant arthritis), the expression of tumour necrosis factor α (TNF-α) was inhibited and this led to a reduction in synovial hyperplasia and joint swelling with maintenance of joint integrity [31]. Similar results were obtained in an autoantibody-mediated murine model [32]. However, no study has looked at the effect of these inhibitors on cartilage. Here, we show for the first time that HDAC inhibitors repress the expression of several members of the metalloproteinase family in chondrocytes and block cartilage destruction in an *ex vivo *model system. Hence, inhibition of HDAC activity offers a potential therapeutic strategy to prevent cartilage destruction in the arthritides.

## Materials and methods

### Cell culture

SW1353 human chondrosarcoma cells (ATCC, USA) were routinely cultured in DMEM (Invitrogen, Paisley, UK) containing 10% fetal bovine serum (Invitrogen), 2 mM glutamine, 100 IU/ml penicillin, and 100 μg/ml streptomycin. Serum-free conditions used identical medium without fetal bovine serum. For assays, cells were grown to confluency, then starved of serum for 24 hours before the addition of IL-1α (R&D Systems) (5 ng/ml) and oncostatin M (OSM) (R&D Systems) (10 ng/ml) in the absence or presence of HDAC inhibitors (TSA, 50 to 500 ng/ml, and NaBy, 1 to 10 mM) (Calbiochem, Nottingham, UK). Experiments were performed in six-well plates (Nunc, Fisher Scientific, Loughborough, UK) with all conditions in duplicate or triplicate. To obtain primary human chondrocytes, fresh human articular cartilage samples (from patients undergoing hip or knee replacement surgery at the Norfolk and Norwich University Hospital) were digested overnight in DMEM containing 2 mg/ml of collagenase Type 1A (Sigma, Poole, UK). The resulting cells were washed with PBS, resuspended in DMEM containing 10% FCS and antibiotics as above, and then plated at 1 × 10^6 ^cells in 75-cm^2 ^flasks. At confluence, cells were passaged and replated at 1:2 dilution.

### RNA isolation and synthesis of cDNA

RNA was isolated from monolayer cultures using Trizol reagent (Invitrogen). cDNA was synthesised from 1 μg of total RNA using Superscript II reverse transcriptase (Invitrogen) and random hexamers in a total volume of 20 μl according to the manufacturer's instructions. cDNA was stored at -20°C until use in downstream PCR.

### RT-PCR

For quantitative real-time PCR, sequences and validation for *MMP *and *TIMP *primers and probes are as previously described [33] and so are *ADAMTS *primers and probes [34]. In order to control against amplification of genomic DNA, primers were placed within different exons close to an intron/exon boundary with the probe spanning two neighbouring exons where possible. BLAST searches for all the primer and probe sequences were also conducted to ensure gene specificity. The 18 S ribosomal RNA gene was used as an endogenous control to normalise for differences in the amount of total RNA present in each sample; 18 S rRNA primers and probe were purchased from Applied Biosystems (Warrington, UK).

Relative quantification of genes was performed using the ABI Prism 7700 sequence detection system (Applied Biosystems) in accordance with the manufacturer's protocol. PCR reactions contained 5 ng of reverse transcribed RNA (1 ng for 18 S analyses), 50% TaqMan 2X Master Mix (Applied Biosystems), 100 nM of each primer, and 200 nM of probe in a total volume of 25 μl. Conditions for the PCR reaction were 2 min at 50°C, 10 min at 95°C, and then 40 cycles each consisting of 15 s at 95°C and 1 min at 60°C.

Conventional RT-PCR for collagen and aggrecan expression was as previously described [35].

### Cartilage degradation assay

Bovine nasal cartilage was cultured as previously described [36]. Briefly, discs (approximately 2 mm in diameter by 1 to 2 mm thick) were punched from bovine nasal septum cartilage; three discs per well in a 24-well plate were incubated overnight in control, serum-free medium (DMEM containing 25 mM HEPES, 2 mM glutamine, 100 μg/ml streptomycin, 100 IU/ml penicillin, 2.5 μg/ml gentamicin, and 40 u/ml nystatin). Fresh control medium with or without test reagents (each condition in quadruplicate) was then added (day 0). Cartilage was incubated until day 7 and supernates were harvested and replaced with fresh medium containing the same test reagents as day 0. On day 14, supernates were harvested and the remaining cartilage was digested with papain. The viability of cartilage explants was assessed by measurement of lactate dehydrogenase (LDH) in the conditioned medium (CytoTox 96 assay, Promega, Southampton, UK). Hydroxyproline release was assayed as a measure of collagen degradation [37], and glycosaminoglycan release was assayed as a measure of proteoglycan degradation [36]. Collagenase activity was determined by the ^3^H-acetylated collagen diffuse fibril assay using a 96-well plate modification [38] and a standard curve and appropriate sample dilutions; one unit of collagenase activity degraded 1 μg of collagen per minute at 37°C. APMA (4-aminophenylmercuric acetate) was used to activate procollagenases [38]. Statistical analysis was performed using Student's *t*-test.

### Gelatin zymography

Samples were electrophoresed under nonreducing conditions by SDS–PAGE in 10% polyacrylamide gels copolymerised with 1% gelatin. Gels were washed vigorously twice for 15 min in 2.5% Triton X-100 to remove SDS, then incubated overnight in 50 mM Tris/HCl, pH7.5, 5 mM CaCl_2 _at 37°C. Gels were then stained with Coomassie brilliant blue. Parallel gels were incubated in buffers containing either 5 mM EDTA or 2 mM 1,10-phenanthroline to show that lysis of gelatin was due to metalloproteinase activity.

### Western blotting

Samples of conditioned culture medium were precipitated with an equal volume of ice-cold 10% w/v trichloroacetic acid. Precipitates were resuspened in loading buffer and electrophoresed under reducing conditions by SDS–PAGE in 10% polyacrylamide gels. Proteins were then transferred to an Immobilon P membrane (Millipore, Watford, UK) and probed with either rabbit anti-(human MMP-1), [39], sheep anti-(human MMP-1) [40], or sheep anti-(human MMP-13) [40].

## Results

### Histone deacetylase inhibitors block cartilage resorption and concurrently decrease collagen- and gelatin-degrading proteolytic activities

**Figure 1:**
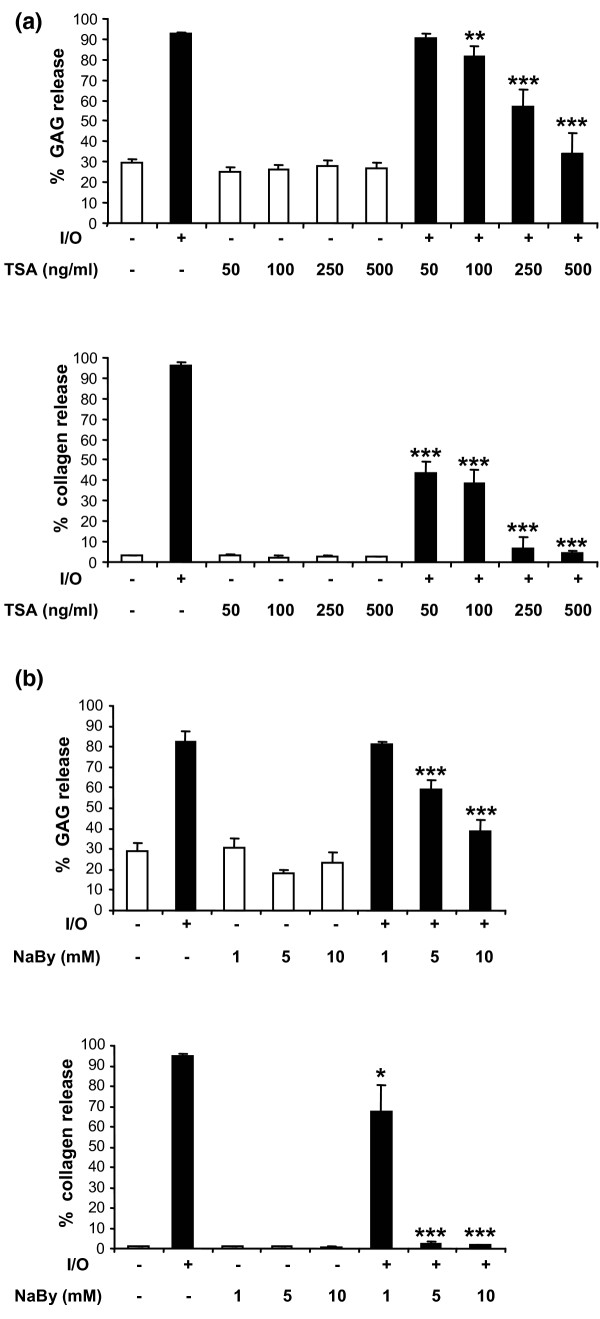
Histone deacetylase inhibitors block cartilage glycosaminoglycan and collagen loss induced by IL-1α/OSM. Bovine nasal cartilage discs were cultured in the presence or absence of I/O (a combination of IL-1α and oncostatin M (OSM)) and a histone deacetylase inhibitor, either **(a) **I/O (1 ng/ml IL-1α, 10 ng/ml OSM) with trichostatin A (TSA) or **(b) **I/O (0.2 ng/ml IL-1α, 2 ng/ml OSM) with sodium butyrate (NaBy). Cartilage was incubated until day 7 and supernates were harvested and replaced with fresh reagents until day 14. Glycosaminoglycan (GAG) release is shown as at day 7 and was assayed using the dimethylmethylene blue method. Collagen release is shown as at day 14 and was measured using an assay for hydroxyproline. Viability was assessed by measuring lactate dehydrogenase in the conditioned medium. Assays were performed at least twice using quadruplicate samples; means ± standard deviations are represented. *P < 0.05, **P < 0.01, ***P < 0.001.

The combination of IL-1α and OSM has previously been shown to reproducibly and potently induce cartilage proteoglycan and collagen proteolysis both *in vitro *and *in vivo *[41,42]. The addition of TSA or NaBy to bovine nasal cartilage explant culture stimulated to resorb with IL-1α and OSM caused a dose-dependent (50 to 500 ng/ml TSA, 1 to 10 mM NaBy) inhibition of both proteoglycan and collagen release (at day 7 and 14 respectively) (Fig. 1). TSA is reported to have an IC_50 _(median inhibitory concentration) in the nanomolar range (50 ng/ml = 165 nM), but this does vary depending upon the HDAC and assay used (e.g. [43]); NaBy is reported to have an IC_50 _in themillimolar range. The need for TSA, a hydroxamate, to penetrate the highly negatively charged cartilage matrix will also raise the effective IC_50 _in the cartilage explant assay. The time points of medium collection, days 7 and 14, are those at which release of, respectively, proteoglycan and collagen are reproducibly close to 100%, since proteoglycan release is an earlier event in cartilage degradation. At these time points, proteoglycan release showed less sensitivity to HDAC inhibitors than collagen release, behaviour that may reflect its more rapid kinetics. Indeed, in a preliminary experiment at day 3, where IL-1α/OSM-induced proteoglycan release was approximately 50%, an increased sensitivity to HDAC inhibitors was seen (data not shown). Lactate dehydrogenase release, used as a measure of toxicity, was no greater in the presence of TSA or NaBy (at any concentration) than in the comparator control cultures (i.e. either without any addition or treated with IL-1α/OSM) at either day 7 or day 14; furthermore, no dose-dependent effects of TSA or NaBy on the release of lactate dehydrogenase were observed (data not shown).

**Figure 2:**
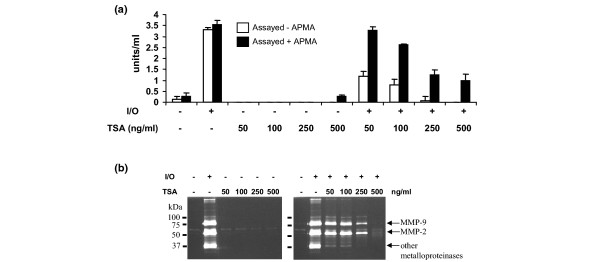
HDAC inhibitors decrease collagenolytic and gelatinolytic activity from bovine nasal explants and block collagenase activation. Conditioned media from cartilage assays (day 14) as in Fig. 1a were assayed **(a) **for collagenase activity in the presence or absence of 0.67 mM APMA, an activator of procollagenases (means ± standard errors of the mean), and **(b) **for gelatinase activity, using gelatin zymography. APMA, aminophenylmercuric acetate; HDAC, histone deacetylase; I/O, a combination of IL-1α and OSM; MMP, matrix metalloproteinase; OSM, oncostatin M; TSA, trichostatin A.

Figure 2a shows collagenase activity at day 14 in the absence or presence of TSA, assayed in the conditioned medium from the explant assay discussed above. Treatment with IL-1α and OSM increased collagenase activity in the medium, and all collagenases were in the active form (since the addition of the procollagenase activator APMA did not lead to an increase in activity). The additional presence of TSA at the lowest dose (50 ng/ml) decreased the level of active collagenase, whereas total collagenase was unchanged; that is, the percentage of collagenase that was active was decreased (since the addition of APMA led to increased activity in the assay, demonstrating the presence of procollagenases). With increasing dose, TSA decreased the level of both active and total collagenase; that is, the total amount of collagenase in the medium was decreased and the percentage of this enzyme(s) that was activated also decreased. Similar results were obtained using NaBy (data not shown).

Figure 2b shows a gelatin zymogram of the day-14 medium cartilage-explant-conditioned medium in the absence or presence of TSA. Unstimulated explants produce a low constitutive level of gelatinolytic activity, which was probably due to proMMP-2. The addition of IL-1α and OSM induced three major gelatinolytic activities, which ran as poorly resolved doublets (all activities shown were blocked by metalloproteinase inhibitors EDTA and 1,10-phenanthroline, and were therefore due to the action of metalloproteinases; see Materials and methods). The largest of these probably equates to bovine MMP-9 (both pro- and active); there was an induction and activation of MMP-2 and an induction of a lower-molecular-weight activity that may represent collagenases MMP-1 and MMP-13, but could include other MMPs, many of which have at least some activity against gelatin. Both of the collagenases, and particularly MMP-13, have gelatinolytic activity [44,45], and this would therefore be in agreement with the induction of collagenase activity shown in Fig. 2. TSA at the lowest dose (50 ng/ml) caused a marked reduction in the lowest-molecular-weight activity, while increasing doses reduced the activities of all the gelatinolytic enzymes to background levels.

### Histone deacetylase inhibitors modulate MMP gene expression

**Figure 3:**
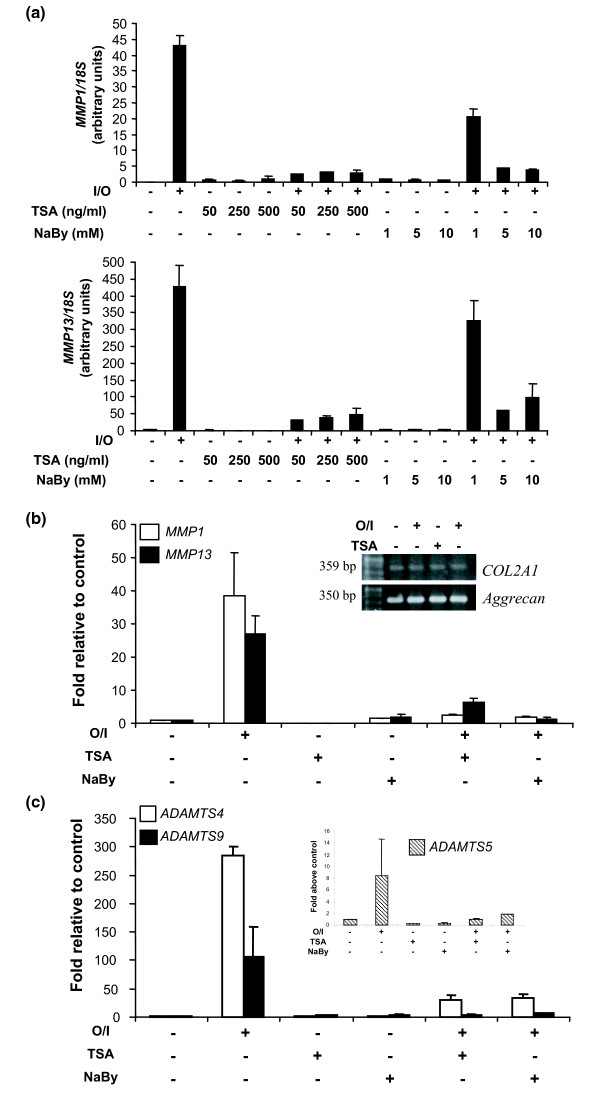
Histone deacetylase inhibitors abrogate IL-1α/OSM-induced expression of key metalloproteinase genes. Cells were starved of serum for 24 hours before stimulation with I/O, a combination of IL-1α (5 ng/ml) and oncostatin M (OSM) (10 ng/ml) for 6 hours in the absence or presence of either trichostatin A (TSA) (**(a) **as shown or **(b, c) **500 ng/ml) or NaBy ((a) as shown; (b, c) 10 mM). Total RNA was isolated and subjected to quantitative RT-PCR for expression of the genes (a, b) *MMP1 *and *MMP13 *and (c) *ADAMTS4*, *ADAMTS5*, and *ADAMTS9*. Data were normalised to the 18 S rRNA housekeeping gene; means and ranges are plotted. Absolute numbers are primer/probe-set-dependent and so cannot be compared between genes. (a) SW1353 chondrosarcoma cells; (b, c) primary human chondrocytes; (b) (inset) expression of *COL2A1 *and *aggrecan *by conventional RT-PCR. I/O, O/I, a combination of IL-1α and OSM; NaBy, sodium butyrate.

Using the SW1353 chondrosarcoma cell line as a model in which to look at the regulation of metalloproteinase and TIMP gene expression, we profiled the expression of all *MMP*s, *ADAMTS*s, and *TIMP*s in cells stimulated with IL-1α and OSM in the absence or presence of HDAC inhibitors at the doses used for the cartilage explant experiments above. Figure 3a shows typical responses for genes induced by IL-1α and OSM. A number of genes – *MMP1*, *MMP3*, *MMP7*, *MMP8*, *MMP10*, *MMP12*, *MMP13*, *ADAMTS4*, and *ADAMTS9 *– were robustly induced by the combination of IL-1α and OSM (though both *ADAMTS4 *and *ADAMTS5 *were expressed only at low levels in this cell line, with *ADAMTS5 *showing a weak induction with IL-1α and OSM). Of these induced genes, including *ADAMTS5*, all but *ADAMTS4 *showed repression by both TSA and NaBy. *ADAMTS4*, while strongly induced by IL-1α and OSM, was not repressed by either HDAC inhibitor in this cell line. The expression of a number of genes (*MMP2*, *MMP9*, *MMP16*, and *MMP19*; *ADAMTS1*, *ADAMTS2*, *ADAMTS7*, *ADAMTS12*, *ADAMTS13*, and *ADAMTS20*; *TIMP3*) was unaffected by the HDAC inhibitors, whereas the expression of several genes was induced by HDAC inhibitors alone (*MMP17*, *MMP23*, *MMP28*; *ADAMTS15 *and *ADAMTS17*; *TIMP2*). The varying response to HDAC inhibitors across the gene families also affirms that the compounds are not simply showing a nonspecific toxicity.

In order to verify that the effects of HDAC inhibitors were not specific to the SW1353 cell line, we undertook a similar experiment on a subset of genes, using primary articular chondrocytes isolated from both knee and hip joint (i.e. from two different donors). *MMP1 *and *MMP13*, the two major specific collagenases, were strongly induced by IL-1α and OSM, and this induction was repressed by both TSA (500 ng/ml) and NaBy (10 mM) (Fig. 3b). *MMP8 *was expressed at much lower levels in these cells but followed the same pattern of responses (data not shown). The IL-1α and OSM induction of *MMP3 *gene expression was only poorly repressed by HDAC inhibitors in the primary chondrocytes (data not shown). In these cells, *ADAMTS4*, *ADAMTS5*, and *ADAMTS9 *were all induced by IL-1α and OSM and the induction was repressed by HDAC inhibitors (Fig. 3c). These primary chondrocytes, although grown in monolayer culture, still express type II collagen and aggrecan at the passage at which this experiment was performed.

### Histone deacetylase inhibitors repress MMP protein expression and activity

**Figure 4:**
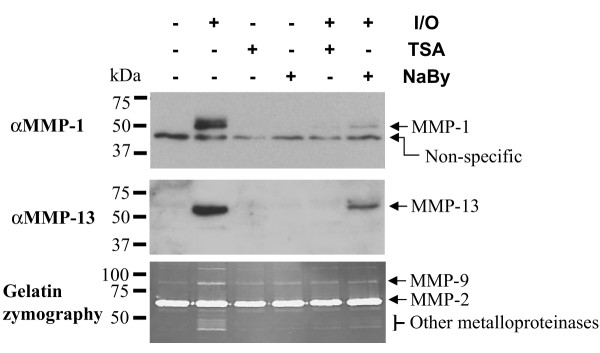
Histone deacetylase inhibitors repress matrix metalloproteinase (MMP) protein expression and activity. Cells were starved of serum for 24 hours before stimulation with I/O, a combination of IL-1α (5 ng/ml) and oncostatin M (10 ng/ml), for 24 hours in the absence or presence of trichostatin A (TSA) (500 ng/ml) or sodium butyrate (NaBy) (10 mM). Conditioned media were subjected to western blot analysis using a rabbit anti-(human MMP-1) antibody or a sheep anti-(human MMP-13) antibody or gelatin zymography, as described in Materials and methods.

In order to ascertain if changes at the level of steady-state mRNA are mirrored at the protein level, we performed western blots on the conditioned medium of SW1353 cells at a 24-hour time point. Both MMP-1 and MMP-13 proteins were potently induced by treatment with IL-1α and OSM and this induction was repressed by both TSA and NaBy in the same manner as the mRNA (Fig. 4). Two different anti-MMP-1 antibodies (one raised in rabbits [39] and one raised in sheep [40]) cross-react with a protein of slightly lower *M*_r _than MMP-1 in the SW1353-conditioned medium. The identity of this protein is unknown, but its expression has been previously documented [40] (though misinterpreted as that of active MMP-1), it is unaltered by the stimuli used, and it is not present in conditioned medium from primary chondrocytes (data not shown).

Gelatin zymography showed some induction of MMP-9, as well as multiple bands at around the *M*_r _of the collagenases that are induced by IL-1α/OSM and repressed by the additional presence of HDAC inhibitors in this system.

## Discussion

HDAC inhibitors are currently being developed as cancer therapeutics, largely by virtue of their impact upon the cell cycle and apoptosis [30] in transformed cells. However, it is clear that such compounds have pleiotropic effects on gene expression. Conceptually, the action of HDAC inhibitors leading to an increase in histone acetylation should induce expression of susceptible genes, but in fact, many instances of a repression of gene expression have been reported [25-29]. In yeast, the ability of TSA to down-regulate some genes very rapidly (within 15 min of exposure) suggests that HDACs may function as direct transcriptional activators in some instances [25].

The combination of IL-1 and oncostatin M potently induces both cartilage aggrecan and collagen degradation *in vitro *and *in vivo *[41,42] and these factors induce the expression of a number of metalloproteinase genes in chondrocyte cell lines [46]. The addition of either of two chemically distinct HDAC inhibitors to cartilage explant cultures blocks IL-1/OSM-induced cartilage catabolism with a decrease in collagenolytic activity in the conditioned culture medium. TSA and NaBy themselves do not directly inhibit collagenase activity, and it therefore seemed likely that they were altering expression of genes encoding the metalloproteinases or their inhibitors. Using SW1353 chondrosarcoma cells, which are known to respond to IL-1/OSM [40], and primary chondrocytes, real-time RT-PCR gene profiling showed that the expression of a number of *MMP *and *ADAMTS *genes was robustly induced by IL-1/OSM and repressed by HDAC inhibitors. In SW1353 cells, *MMP2 *is not induced by IL-1/OSM nor altered by HDAC inhibitors; *MMP9 *is weakly induced by IL-1/OSM and this induction is repressed by HDAC inhibitors. In primary chondrocytes, *MMP2 *expression is induced approximately twofold to fourfold by IL-1/OSM, but is not then repressed by HDAC inhibitors. This is in marked contrast to the zymography data from cartilage explants and suggests a role for cell–matrix interactions in mediating the effects of IL-1/OSM on these gelatinase genes.

Previous studies have shown that TSA represses *MMP2 *expression in mouse 3T3 fibroblasts but not in human HT1080 fibrosarcoma cells [47,48], showing that the effects of HDAC inhibitors on MMP expression are specific to cell type and potentially to species. In primary chondrocytes, the effects of HDAC inhibitors on the collagenases (*MMP1*, *MMP8*, *MMP13*) mirrored that seen in the SW1353 cell line; however, *MMP3*, though strongly induced by IL-1/OSM in primary chondrocytes, was not significantly repressed by HDAC inhibitors. The ability of HDAC inhibitors to repress *MMP *expression at the mRNA level is reiterated at the protein level, as we have shown for MMP-1 and MMP-13. In primary chondrocytes, the aggrecanases *ADAMTS4*, *ADAMTS5*, and *ADAMTS9 *were also strongly induced by IL-1/OSM and were repressed by HDAC inhibitors. In SW1353 cells, both *ADAMTS4 *and *ADAMTS5 *genes are expressed at a very low level and are therefore difficult to measure; *ADAMTS9*, however, is expressed robustly, is induced by IL-1/OSM, and is repressed by HDAC inhibitors, with a pattern similar to that shown for *MMP1 *and *MMP13 *in Fig. 3a. This repression of aggrecanase gene expression is consistent with the ability of HDAC inhibitors to inhibit cartilage glycosaminoglycan release as shown in Fig. 1.

HDAC inhibitors appear to affect not only the expression of collagenolytic and gelatinolytic MMPs, but also their activation (Fig. 2). It is known that activation of procollagenases is a key control point in cartilage resorption and this can be mediated by cascades within the MMP family (e.g. MMP-3 can activate procollagenases) or via the action of other enzyme families (e.g. plasmin, a serine proteinase) [49,50]. Therefore, it is likely that HDAC inhibitors repress the expression of one or more key procollagenase activators in cartilage; study of, for example, plasminogen or plasminogen activator expression might be informative.

Since almost all metalloproteinase genes that are robustly induced by IL-1/OSM are then repressed by the further addition of HDAC inhibitors, a likely explanation is the ability of HDAC inhibitors to interfere with IL-1/OSM signalling. Since these cytokines are proinflammatory mediators, action via nuclear factor κB is one possibility; however, the literature shows that TSA actually potentiates signalling through this pathway [51,52]. OSM, an IL-6 family cytokine, signals through the STAT pathway; recent reports show that HDAC activity plays an essential role in at least STAT1 signalling, and that TSA can therefore abrogate STAT1-induced gene expression [53,54]. We (TEC and co-workers) have previously reported that at least STAT3 signalling indirectly mediates the ability of IL-1/OSM to induce *MMP1 *gene expression [55]. Dissecting the pathways that mediate the impact of HDAC inhibitors on induction of metalloproteinase genes by IL-1/OSM will therefore be one focus of our future work.

Two previous reports using the rodent models of rheumatoid arthritis (rat adjuvant arthritis and murine autoantibody-mediated arthritis) showed that HDAC inhibitors (TSA, phenylbutyrate, or FK228) block proliferation of cultured synovial fibroblasts with accompanying up-regulation of cell cycle inhibitors (p16^INK4 ^and p21^Cip1^) [31,32]. *In vivo*, this was mirrored with inhibition of synovial hyperplasia and pannus formation, leading to abrogation of cartilage destruction in the models. Interestingly, the HDAC inhibitors also repressed expression of TNF-α and/or IL-1 in synovial tissue. These reports suggest that HDAC inhibitors may represent a new class of compounds for treatment of rheumatoid arthritis [31,32]. Anti-inflammatory properties of another HDAC inhibitor, suberoylanilide hydroxamic acid (SAHA), have also been demonstrated *in vitro *and *in vivo*, via the suppression of proinflammatory cytokines such as TNF-α and IL-1 [56,57].

All of these papers suggest a major effect of HDAC inhibitors in repressing the production of proinflammatory cytokines. Our current data show for the first time that HDAC inhibitors can also function as potent repressors of key metalloproteinase expression in cartilage and chondrocytes and thus block cartilage breakdown. This suggests they may have wider therapeutic use outside of just the inflammatory arthritides, as chondroprotective agents. It should be underlined that where the action of HDAC inhibitors is to repress the induced expression of *MMP *or *ADAMTS *genes, this expression is pushed back to control levels but not to zero. This may be important in any therapeutic use of HDAC inhibitors, since normal connective tissue turnover may therefore be unimpaired (though it should also be noted that some *MMP *and *ADAMTS *genes are induced by HDAC inhibitors). Our future work will identify the HDAC(s) that have an effect on metalloproteinase expression and identify the mechanism by which this occurs. This has the potential to allow the design and use of compounds specific for one HDAC (or a small number of HDACs), which may be crucial in avoiding toxicity *in vivo*.

## Conclusion

HDAC inhibitors can repress the expression and activity of matrix-degrading proteinases in chondrocytes and cartilage. These compounds, in preclinical development as chemotherapeutic agents, also have strong potential as chondroprotective agents.

## Abbreviations

ADAMTS = a disintegrin and metalloproteinase domain (ADAM) with thrombospondin motifs; APMA = 4-aminophenylmercuric acetate; DMEM = Dulbecco's modified Eagle's medium; ECM = extracellular matrix; FCS = fetal calf serum; HDAC = histone deacetylase; IC_50 _= median inhibitory concentration; IL = interleukin; MMP = matrix metalloproteinase; NaBy = sodium butyrate; OSM = oncostatin M; PCR = polymerase chain reaction; RT = reverse transcriptase; TIMP = tissue inhibitor of metalloproteinases; TNF = tumour necrosis factor; TSA = trichostatin A.

## Competing interests

IMC, DAY, and TEC have filed a patent relating to the contents of this manuscript.

## Authors' contributions

DAY helped conceive the study, designed and carried out cell experiments, carried out some of the real-time RT-PCR experiments, and helped to draft the manuscript. RLL carried out cartilage resorption assays and assessed toxicity of the HDAC inhibitors in this system. CJP designed real-time PCR primer probe sets and carried out some of the real-time RT-PCR. DJ carried out proteinase activity assays associated with the study. LK carried out real-time RT-PCR associated with the cell experiments. DRE designed and validated the real-time PCR methodology and helped to draft the manuscript. TEC designed the cartilage resorption assays and helped to draft the manuscript. IMC helped conceive, design, and coordinate the study, carried out some cell experiments, and helped to draft the manuscript. All authors read and approved the final manuscript.
